# Reverse Takotsubo Cardiomyopathy Complicated by Neurogenic Pulmonary Edema in Aneurysmal Subarachnoid Hemorrhage: A Case Report

**DOI:** 10.7759/cureus.117068

**Published:** 2026-09-28

**Authors:** Mohammed Mansoor Nainthramveetil, Ahmed Elyas, Shajahan Idayathulla, Umme Nashrah, Nissar Shaikh

**Affiliations:** 1 Department of Surgical Intensive Care, Hamad Medical Corporation, Doha, QAT; 2 Department of Cardiology and Cardiovascular Surgery, Heart Hospital, Hamad Medical Corporation, Doha, QAT; 3 Department of Anesthesiology, Hamad Medical Corporation, Doha, QAT; 4 Department of Medicine, Deccan College of Medical Sciences, Hyderabad, IND

**Keywords:** aneurysmal subarachnoid hemorrhage, catecholamine surge, neurocardiogenic injury, neurogenic pulmonary edema, reverse takotsubo cardiomyopathy, stent-assisted coiling

## Abstract

Takotsubo cardiomyopathy (TTC) is a recognized and potentially severe cardiac complication in patients with aneurysmal subarachnoid hemorrhage (aSAH). Reverse TTC (rTTC), a rare variant characterized by basal hypokinesis with apical sparing, is infrequently reported in this setting. The coexistence of rTTC with neurogenic pulmonary edema has not been previously described and represents a complex neurocardiac interaction that can significantly worsen outcomes.

We report the case of a previously healthy 30-year-old man who presented with high-grade aSAH secondary to rupture of an anterior communicating artery aneurysm. His course was complicated by neurogenic pulmonary edema, refractory hypoxemia, hemodynamic instability, and rTTC. Management required early airway protection, bronchoscopy, vasopressor support, and complex stent-assisted endovascular coiling. Echocardiography demonstrated reduced left ventricular ejection fraction with basal hypokinesis and apical sparing, consistent with rTTC. Careful hemodynamic management was essential to balance cerebral perfusion against myocardial dysfunction.

With multidisciplinary critical care and structured rehabilitation, the patient achieved favorable neurological and functional recovery. This case highlights that rTTC in aSAH is not a benign condition and may be associated with increased morbidity when combined with neurogenic pulmonary edema due to massive catecholamine surge. Early recognition using bedside echocardiography, prompt aneurysm securing, and balanced hemodynamic support are critical for improving outcomes.

## Introduction

Reverse Takotsubo cardiomyopathy (rTTC) is a rare variant of stress cardiomyopathy, also known as broken-heart syndrome. Classic Takotsubo cardiomyopathy (TTC) is characterized by apical ballooning with basal hypercontractility. rTTC shows the opposite pattern: basal hypokinesis or akinesis with apical sparing [1,2].

TTC accounts for about 2% of troponin-positive presentations with suspected acute coronary syndrome [1,2]. Among patients with TTC, the reverse variant is uncommon. Published series report 1%-23%, and the International Takotsubo Registry found the reverse pattern in 2.2% of 1,750 patients [2]. rTTC is reported more often in younger patients and in association with neurological disease [2].

Aneurysmal subarachnoid hemorrhage (aSAH) can produce a sudden catecholamine surge. That surge may stun the myocardium and increase pulmonary capillary permeability, so cardiac dysfunction and neurogenic pulmonary edema can occur together [3,4]. The clinical picture can resemble acute coronary syndrome or aspiration pneumonia: unexpected hypoxemia, a modest troponin rise, and shock. The electrocardiogram may show no ischemic ST-segment change. Echocardiography is the practical diagnostic test, because the wall-motion abnormality extends beyond a single coronary territory [5,6].

rTTC after aSAH has been reported only in isolated cases and has rarely been described together with neurogenic pulmonary edema [7,8]. We report a case of World Federation of Neurosurgical Societies grade IV aSAH complicated by neurogenic pulmonary edema and rTTC with a favorable outcome.

## Case presentation

A previously healthy 30-year-old man was found unconscious in his bathroom. Emergency medical services recorded an initial Glasgow Coma Scale of 9 (E3V2M4), which improved to 10 (E3V2M5) (World Federation of Neurosurgical Societies grade IV) [9]. Pupils were 4 mm and briskly reactive. Oxygen saturation was 88% on room air despite normal respiratory effort, which improved to 98% with a non-rebreather mask. He was transported to the emergency department.

Noncontrast computed tomography (CT) of the head showed diffuse bilateral subarachnoid hemorrhage involving the basal cisterns and sylvian fissures, with unremarkable ventricles (modified Fisher grade 3) [10], highly suggestive of a ruptured anterior communicating (ACom) artery aneurysm. After CT, the patient became agitated with worsening hypoxemia (oxygen saturation 88% on a nonrebreather mask) and was electively intubated. Postintubation, he developed refractory hypoxia despite high fraction of inspired oxygen (FiO_2_) and positive end-expiratory pressure (PEEP). Chest radiographs demonstrated bilateral pulmonary opacities, initially more prominent on the left, progressing to involve the right side, consistent with neurogenic pulmonary edema or aspiration pneumonitis (Figures 1, 2). Endovascular coiling was deferred by the anesthesiologist because of hemodynamic instability and hypoxemia. Figure 2 shows the follow-up chest radiograph showing progression and persistence of bilateral infiltrates.

**Figure 1:**
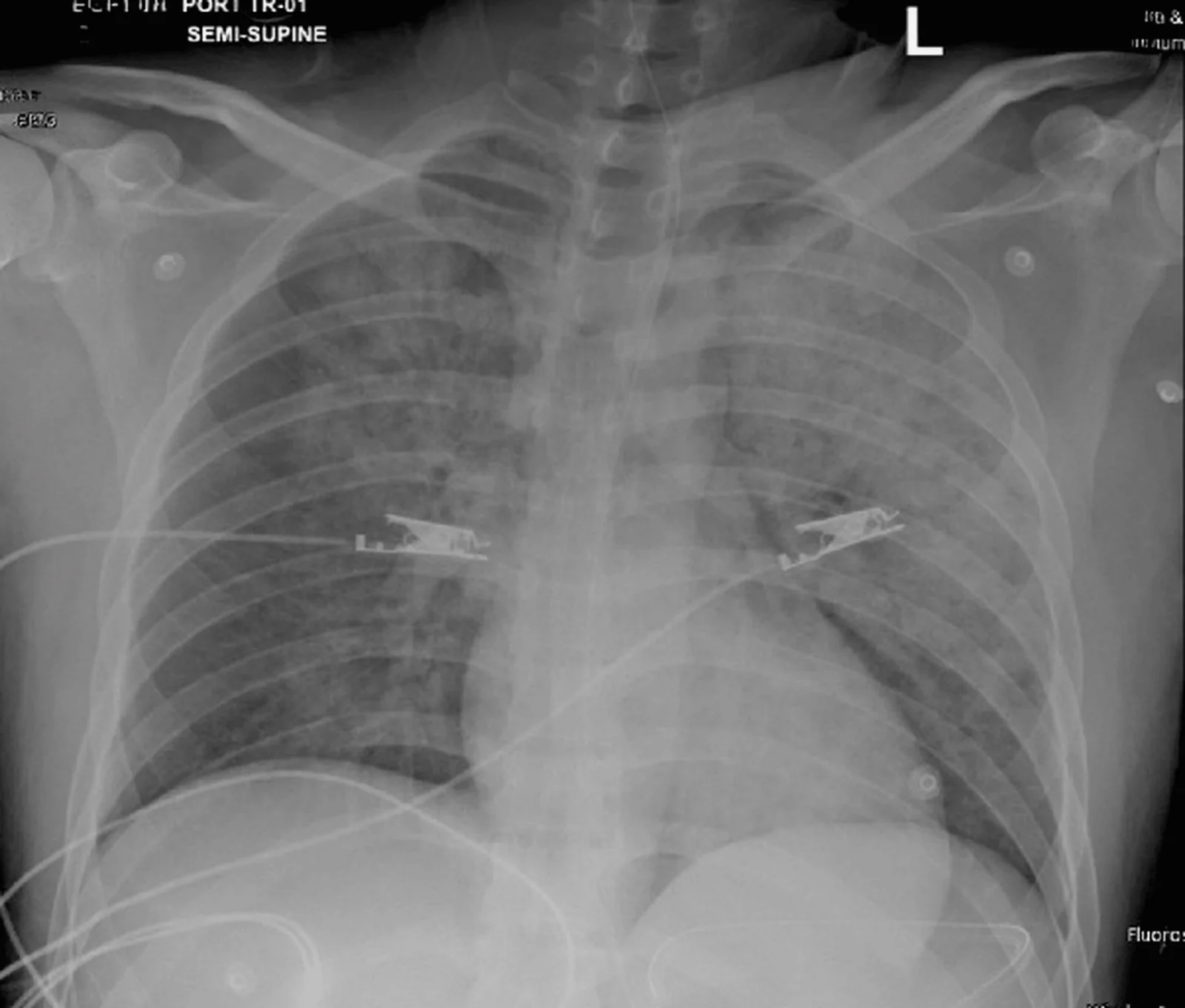
Initial chest radiograph showing bilateral pulmonary opacities suggestive of aspiration pneumonitis or neurogenic pulmonary edema

**Figure 2:**
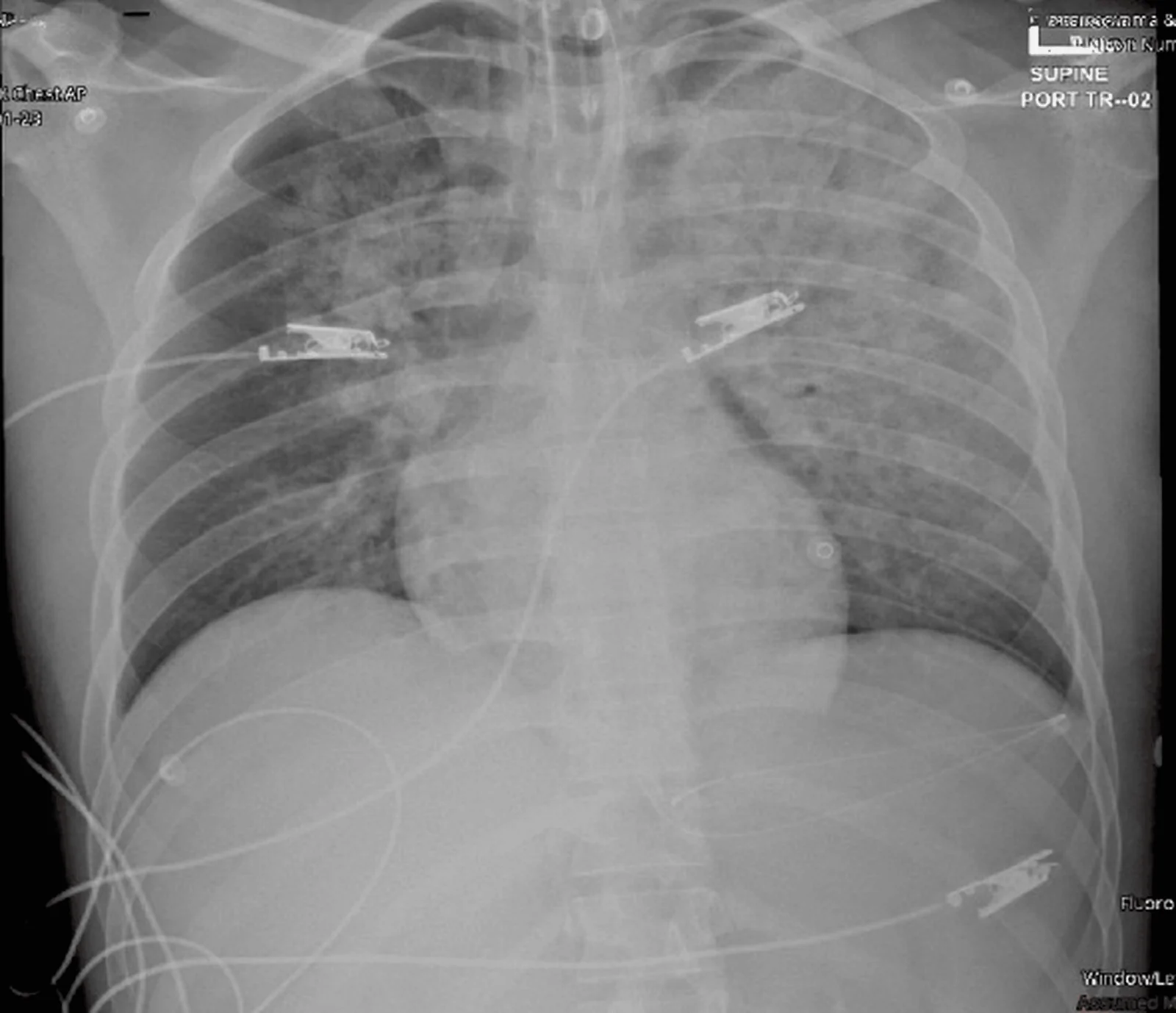
Follow-up chest radiograph showing progression and persistence of bilateral infiltrates

Surgical intensive care unit admission and initial management

On surgical intensive care unit arrival, the patient was mechanically ventilated (FiO_2_ 1.0, PEEP 10 cmH_2_O) and required norepinephrine (0.7 mcg/kg/minute) through a peripheral line. Point-of-care ultrasound showed a collapsed inferior vena cava with fair cardiac contractility. A right internal jugular central line and radial arterial line were placed, and fluid was given. Norepinephrine was reduced to 0.2 mcg/kg/minute with mean arterial pressure (MAP) about 70 mmHg. Until the aneurysm was secured, systolic blood pressure was kept below 140 mmHg. Cisatracurium was started for ventilator dyssynchrony. Nimodipine and levetiracetam were commenced.

On arrival, the PaO_2_/FiO_2_ ratio was 60, with lactate 3.40 mmol/L. Immediately before bronchoscopy, the PaO_2_/FiO_2_ ratio was 162 and lactate 1.60 mmol/L. Lactate improved after fluid resuscitation and restoration of perfusion. Bronchoscopy revealed hyperemic mucosa without aspirated material. FiO_2_ then fell to 0.5 with PEEP 10 cmH_2_O. After bronchoscopy, the same evening, the PaO_2_/FiO_2_ ratio was 423 and lactate 1.50 mmol/L. Metabolic acidosis also improved.

Neurointervention

Digital subtraction angiography demonstrated a complex 5.0 x 5.5 mm ACom aneurysm with a 3 mm neck incorporating the left A2 segment and a hypoplastic right A1. Balloon-assisted coiling was attempted, but coil protrusion necessitated stent-assisted reconstruction with a Neuroform Atlas 3.0 x 24 mm stent (Stryker Corporation, USA). Complete occlusion was achieved with preserved distal flow. The patient received an eptifibatide bolus of 6.2 mL followed by an infusion at 11 mL/hour for 24 hours, then ticagrelor 180 mg followed by 90 mg twice daily for three months and aspirin 100 mg daily. Postprocedure CT showed interval extension of blood into the occipital horns and fourth ventricle (modified Fisher grade 4) without significant midline shift.

Diagnosis of rTTC

High-sensitivity troponin-T peaked at 730 ng/L (later declining to 255 ng/L) with N-terminal pro-B-type natriuretic peptide of 3,387 pg/mL. Table 1 shows key laboratory, gas-exchange, and hemodynamic values with reference ranges.

**Table 1:** Laboratory, oxygenation, and hemodynamic values with reference ranges

Parameter	Result	Reference range
Scene SpO_2_ on air	88%	≥94%-95%
PaO_2_/FiO_2_ on SICU arrival (Day 1; 05:16)	60	>300
Arterial lactate on SICU arrival	3.40 mmol/L	0.36-1.60 mmol/L
PaO_2_/FiO_2_ before bronchoscopy (Day 1; 11:30)	162	>300
Arterial lactate before bronchoscopy	1.60 mmol/L	0.36-1.60 mmol/L
PaO_2_/FiO_2_ after bronchoscopy (Day 1; 19:03)	423	>300
Arterial lactate after bronchoscopy	1.50 mmol/L	0.36-1.60 mmol/L
High-sensitivity troponin-T, peak then repeat	730 then 255 ng/L	<14 ng/L
NT-proBNP	3,387 pg/mL	<125 pg/mL (age <75)
Left-ventricular ejection fraction (Day 1)	48%	≥52%-54% (male, biplane)
Norepinephrine	0.7 µg/kg/minute on arrival; 0.2 after fluid; 0.06 from Day 5; stopped on Day 14	Titrated to MAP 90-110 mmHg after coiling

Transthoracic echocardiography on Day 1 showed an auto-biplane ejection fraction of 48% with basal hypokinesis and apical sparing, consistent with rTTC (Figures 3A, 3B, 4A, 4B).

**Figure 3:**
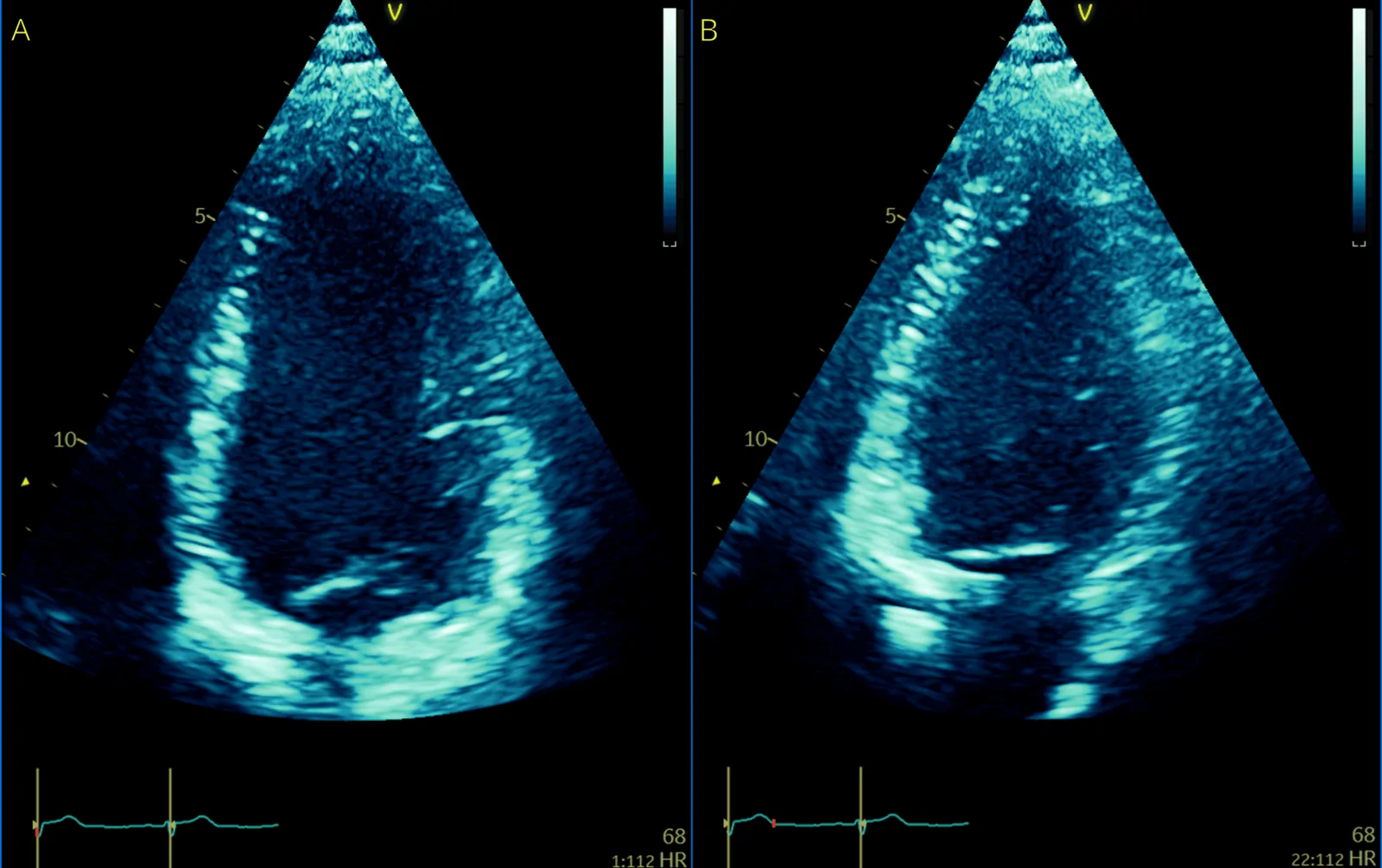
Apical four-chamber views. (A) End-diastole and (B) end-systole, showing basal hypokinesis/akinesis with apical sparing, consistent with reverse Takotsubo cardiomyopathy

**Figure 4:**
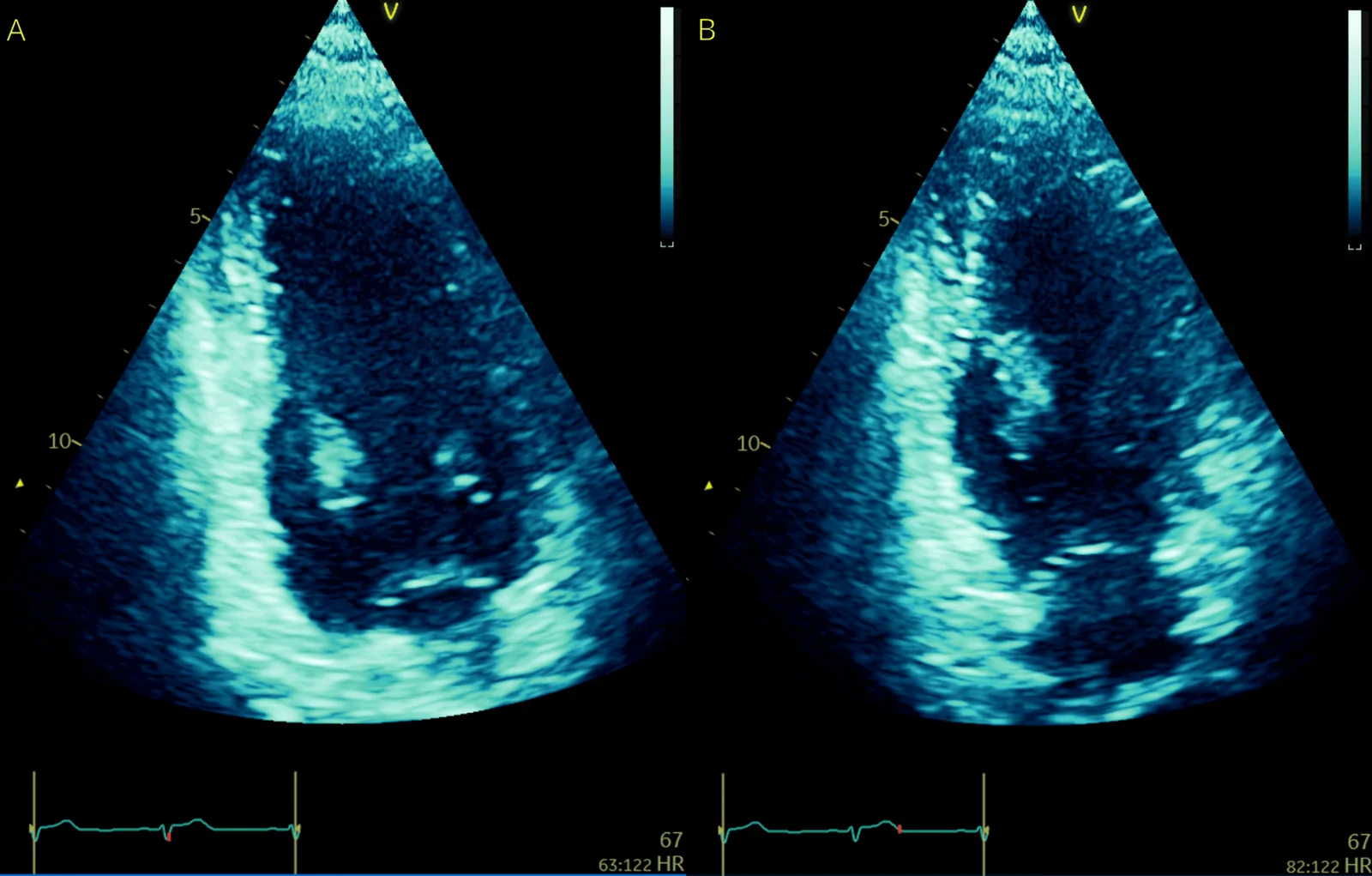
Apical two-chamber views. (A) End-diastole and (B) end-systole, showing basal inferior wall-motion abnormality

The 12-lead electrocardiogram (ECG) showed sinus rhythm with left ventricular hypertrophy but no ischemic ST-T changes (Figure 5).

**Figure 5:**
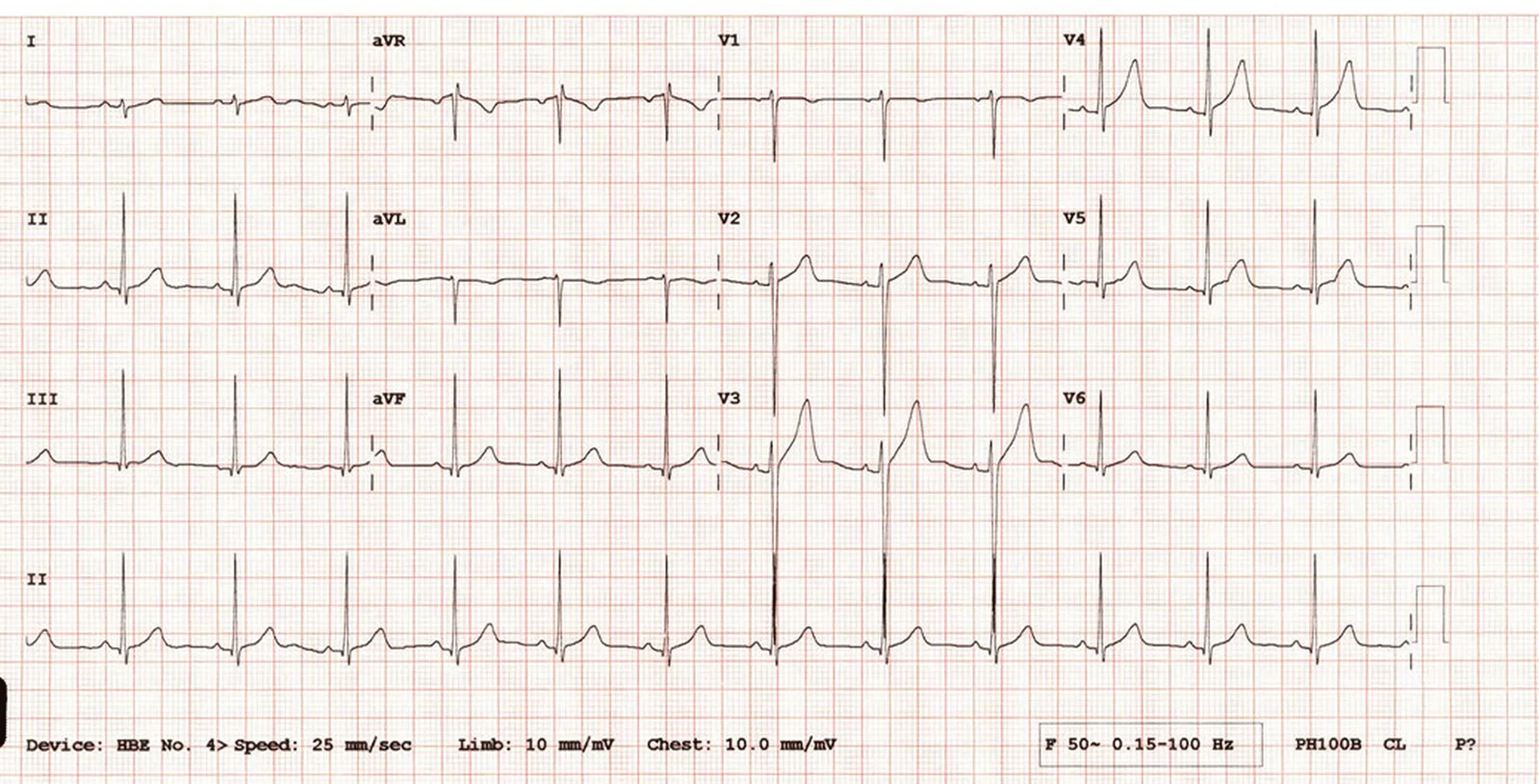
12-lead ECG demonstrating sinus rhythm with left ventricular hypertrophy and no specific ST-T wave changes ECG: electrocardiogram; aVR: augmented vector right; aVL: augmented vector left; aVF: augmented vector foot

After the aneurysm was secured, MAP was targeted at 90-110 mmHg with the lowest effective dose of norepinephrine. Coronary angiography was not performed.

Intensive care unit course and recovery

Oxygenation improved from a PaO_2_/FiO_2_ ratio of 60 on arrival to 423 the same evening. On Day 4, arterial pressure was 145/77 mmHg (MAP about 100 mmHg). Norepinephrine was reduced to 0.06 mcg/kg/minute from Day 5 and was stopped on Day 14. The patient was extubated on Day 6. Serial transcranial Doppler showed resolving vasospasm. CT brain revealed small left temporal and bifrontal ischemic foci without midline shift. Right-sided monoparesis improved gradually. A second echocardiogram was not obtained. The patient was transferred to the neurosurgery ward, breathing room air and hemodynamically stable.

Rehabilitation and discharge

He was admitted to inpatient rehabilitation on Day 21 and discharged home on Day 48. Right-sided strength returned to 5/5, Berg Balance Scale reached 56/56 [11], and he achieved independent ambulation of more than 300 m and stair climbing. Functional Independence Measure rose from 92/126 to 124/126. He was discharged home with a modified Rankin Scale score of 2 [12], independent in activities of daily living, and with no residual cardiac or respiratory limitation. There was no recurrent pulmonary edema, arrhythmia, or shock after vasopressors were stopped.

Discharge medications and follow-up

The patient is followed in outpatient cardiology and neurosurgery clinics, has resumed his job, and remains on aspirin 100 mg daily, ticagrelor 90 mg twice daily (for three months), and levetiracetam 500 mg twice daily. Recovery of left ventricular ejection fraction was inferred clinically and was not documented on a follow-up echocardiogram.

## Discussion

TTC, also known as stress-induced or broken heart syndrome, is a transient, reversible cardiomyopathy associated with significant morbidity in the acute phase. It is increasingly recognized as a form of neurocardiogenic injury in patients with aSAH [3,13]. The overall incidence of TTC is estimated at 1%-3% among patients presenting with suspected acute coronary syndrome, with an annual incidence of 15-30 cases per 100,000 population [1]. In the specific context of aSAH, the incidence is substantially higher, ranging from approximately 4%-8% in symptomatic cases, and echocardiographic evidence of cardiac dysfunction may be detected in up to 20-30% of patients [2,13].

The classical form of TTC is characterized by apical ballooning with hypercontractile basal segments. In contrast, the reverse (or inverted) variant (rTTC) features hypokinesis or akinesis of the basal and mid-ventricular segments with preserved or hyperkinetic apical contractility. This variant is uncommon. Published series report 1%-23% of TTC cases, and the International Takotsubo Registry found the reverse pattern in 2.2% of 1,750 patients [2]. It tends to occur more frequently in younger patients and those with neurological triggers [2]. A review of TTC variants in subarachnoid hemorrhage reported higher plasma epinephrine levels in rTTC than in classic TTC, supporting a catecholamine-mediated mechanism with regional myocardial sensitivity differences [2,4].

The underlying pathophysiology involves a sudden massive catecholamine surge triggered by the acute brain injury, leading to direct myocardial toxicity, microvascular dysfunction, and myocardial stunning [3,4]. This surge also explains the frequent coexistence of neurogenic pulmonary edema through increased pulmonary vascular permeability and left ventricular dysfunction. Although neurogenic pulmonary edema combined with classical TTC has been rarely described in aSAH, the specific association with the reverse variant has not been previously reported to our knowledge. In our patient, the simultaneous occurrence of rTTC and neurogenic pulmonary edema markedly exacerbated hypoxemia and hemodynamic instability, creating a vicious cycle that required aggressive ventilatory and circulatory support [7,8].

Diagnosis in critically ill aSAH patients is challenging because classic symptoms such as chest pain are often masked by altered consciousness. Electrocardiographic changes and modest troponin elevation can mimic acute coronary syndrome, while pulmonary edema may be attributed solely to aspiration or fluid overload. Bedside echocardiography is pivotal, revealing wall motion abnormalities that extend beyond a single coronary artery distribution. The revised Mayo Clinic criteria [5] and the InterTAK Diagnostic Score [6] are valuable tools to differentiate TTC variants from acute coronary syndrome, particularly in settings where urgent coronary angiography may be risky due to neurological instability.

Management of rTTC in aSAH poses unique challenges due to competing priorities: maintaining adequate cerebral perfusion pressure (often requiring vasopressors and induced hypertension) to prevent delayed cerebral ischemia, while avoiding excessive afterload that could worsen myocardial dysfunction [3,13]. In this case, norepinephrine was carefully titrated to the minimal effective dose targeting an MAP of 90-110 mmHg, with close clinical monitoring. Early securing of the ruptured aneurysm via stent-assisted coiling was crucial to prevent rebleeding and allow safe subsequent hemodynamic optimization. Although left ventricular dysfunction in TTC is usually reversible within days to weeks, complications such as cardiogenic shock, arrhythmias, and thromboembolic events can occur [8]. Patients with aSAH-associated TTC, especially severe forms, experience longer intensive care unit stays and higher mortality rates, particularly when complicated by poor-grade hemorrhage or multiorgan involvement [14-16].

This case demonstrates that, despite the initial severity, excellent outcomes are achievable with early recognition using point-of-care echocardiography, prompt aneurysm treatment, lung-protective ventilation, judicious hemodynamic support, and a multidisciplinary approach involving neurointensivists, interventional neuroradiologists, cardiologists, and rehabilitation specialists. A limitation is the absence of follow-up echocardiography. Recovery of left ventricular function was inferred clinically from discontinuation of vasopressors, the absence of recurrent pulmonary edema or shock, and discharge without cardiac limitation.

## Conclusions

rTTC, especially when combined with neurogenic pulmonary edema, represents a rare but serious complication of aSAH. Prompt recognition, echocardiographic confirmation, hemodynamic optimization, and multidisciplinary management can lead to excellent recovery even in complex presentations. In this case, recovery of ejection fraction was inferred clinically because a follow-up echocardiogram was not obtained.
